# Evolution of Superhydrophilic Aluminum Alloy Properties in Contact with Water during Cyclic Variation in Temperature

**DOI:** 10.3390/ma15072447

**Published:** 2022-03-26

**Authors:** Alexander G. Domantovsky, Elizaveta V. Chulkova, Kirill A. Emelyanenko, Konstantin I. Maslakov, Alexandre M. Emelyanenko, Ludmila B. Boinovich

**Affiliations:** 1 A.N. Frumkin Institute of Physical Chemistry and Electrochemistry, Leninsky Prospect 31 Bldg. 4, 119071 Moscow, Russia; doman-alex@yandex.ru (A.G.D.); chulkova_liza@mail.ru (E.V.C.); 9554625@mail.ru (A.M.E.); boinovich@mail.ru (L.B.B.); 2 N.S. Kurnakov Institute of General and Inorganic Chemistry, Leninsky Prospect 31, 119071 Moscow, Russia; 3 Chemistry Department, Lomonosov Moscow State University, 119991 Moscow, Russia; nonvitas@gmail.com

**Keywords:** superhydrophilicity, durability, aluminum hydroxides, thermocycling, alumina

## Abstract

Hydrophilic or superhydrophilic materials in some cases are considered to be potentially icephobic due to a low ice-adhesion strength to such materials. Here, the evolution of the properties of a superhydrophilic aluminum alloy with hierarchical roughness, fabricated by laser processing, was studied in contact with water during prolonged cyclic variation in temperature. It was shown that the chemical interaction of rough alumina with water molecules caused the substitution of the surface oxide by polymorphic crystalline gibbsite or bayerite phases while preserving hierarchical roughness. Due to such substitution, mechanical durability was notably compromised. Thus, in contrast to the superhydrophobic laser-processed samples, the superhydrophilic samples targeted on the exploitation in an open atmosphere as a material with anti-icing properties cannot be considered as the industrially attractive way to combat icing.

## 1. Introduction

Hydrophilic or superhydrophilic materials in some cases are considered potentially icephobic due to low ice-adhesion strength to hydrophilic surfaces at negative ambient temperatures [1]. The main mechanism for this low adhesion is related to the presence of nonfreezing water, which serves as a lubricating interfacial layer. Two mechanisms related to the formation of such a nonfrozen water layer are discussed in the literature. The first one considers the formation of a hydrated water layer on top of hydrophilic polymers, polymer gels, or polyzwitterion brushes, which have a high affinity to water molecules [2,3,4,5]. This hydrated water layer does not crystallize even at a temperature below −50 °C [3].

The second one can be induced by hydrophilic surfaces with nanopores when the surface forces inside the pore cause an alteration in the properties of water by shifting freezing temperatures inside the pore toward low negative values [1,6,7].

Although the idea to use superhydrophilic surfaces––for example as an anti-icing material––in open atmospheric conditions looks attractive, the critical point in development is related to scarce data on the evolution of their properties during cyclic variations in temperature from low negative to high positive. At the same time, the stability of the properties and the nanoporous structure of such material is a prerequisite for preserving the lubricating bound water layer and hence for their use in continuous contact with liquid and solid aqueous atmospheric precipitation.

Herein we present the first study of the evolution of the properties of a superhydrophilic aluminum alloy with hierarchical roughness in contact with water during prolonged cyclic temperature variation and discuss the possibility of applying such material in cold environmental conditions in the presence of atmospheric water.

## 2. Materials and Methods

### 2.1. Sample Preparation

Samples measuring 30 × 20 × 2 mm^3^ were cut from flat coupons (2 mm thick) of aluminum–magnesium alloy AMG2 (LLC Neva-Metal, Saint Petersburg, Russia). According to the supplier’s data, the composition of the alloy (in weight %) is as follows: Al 95.55, Mg 2.9, Mn 0.2, Cr 0.05, Cu 0.1, Fe 0.4, Si 0.4, Ti 0.1, Zn 0.2, and total impurities 0.1. The laser texturing procedure is described elsewhere [8]; specifically, the samples were initially ground and polished with a set of SiC abrasive paper, ultrasonically washed in deionized water, air-dried, and then treated by a nanosecond laser having the following processing parameters: pulse duration 120 ns; pulse frequency, 40 kHz; laser output power, 100 W; laser beam velocity, 1000 mm/s; scanning density, 150 lines/mm; peak fluence, 140 J/cm^2^; and laser beam diameter, (at 1/e^2^ level) 30 μm. Laser treatment was performed under ambient conditions with a relative humidity of ≈40% and a temperature of 20–22 °C using a LaserScan F2 laser system (LLC “Ateko-TM”, Moscow, Russia) equipped with an IR ytterbium fiber laser with a wavelength of 1064 nm. To remove surface micro- and nanoparticles that adhered weakly to the textured surface after laser processing, the samples were ultrasonically washed in deionized water and were superhydrophilic, as will be discussed below.

### 2.2. Thermal Cycling

To study the evolution of the properties of superhydrophilic substrates in contact with an aqueous medium under cyclic variation in temperature, each sample was placed into a polypropylene container with water, and the containers were fixed inside the thermostated bath. The amount of water was fixed and corresponded to 4 mL/cm^2^ of the textured sample’s surface. Our aim was to monitor the wettability, appearance and the chemical and phase composition of the sample’s surface layer during cyclic variation in the water bath temperature from +20 °C to −30 °C. The number of cycles for each sample exceeded 100. We checked the freezing/melting of the water that was in contact with the sample in each temperature cycle with a full cycle time of 180 min. 

After every five cycles the samples were withdrawn from the container, soaked up with a filter paper, and air-dried for 30 min before the wettability evaluation. The water inside the polypropylene container was changed every five cycles.

### 2.3. Hydrophobic Modification of Samples after Thermal Cycling

To find more information about the peculiarities of the texture and its mechanical properties, the samples were hydrophobized by chemisorption of fluorosilane after finalizing the thermal cycling, wetting measurements, and measurements of the elemental and phase composition. For the successful surface binding of hydrophobic molecules, the samples were exposed to vapors of CF_3_(CF_2_)_7_CH_2_O(CH_2_)_3_Si(OCH_3_)_3_ at a temperature of 105 °C inside the sealed cell for 1 h and dried in an oven at 150 °C for 1 h. This heat treatment resulted in the cross-linking of the adjacent adsorbed molecules by siloxane bond formation, as described in [9]. The above modification transformed the samples to superhydrophobic, as will be discussed below.

### 2.4. Surface Characterization

Field-emission scanning electron microscopy (FE-SEM) and energy-dispersive X-ray spectroscopy (EDX) were used to study the morphology and elemental composition of the samples. These studies were performed using a FIB-SEM Nvision 40 workstation (Zeiss, Jena, Germany) equipped with an X-MAX energy-dispersive detector (Oxford Instruments, Abingdon, UK). To inspect the surface topography, the SEM images were recorded in secondary electron (SE) detection mode at accelerating voltages of 2 kV. The EDS spectra were acquired at a 5 kV accelerating voltage.

Furthermore, X-ray photoelectron spectroscopy (XPS) was used to measure the elemental composition of the surface layer and determine the binding states of the elements. The XPS spectra were acquired on an Axis Ultra DLD spectrometer (Kratos Analytical, Manchester, UK) with a monochromatic AlKα radiation source (1486.69 eV, 150 W). Photoelectrons were collected using a semispherical analyzer combined with electrostatic and magnetic lenses. The analyzer axis was perpendicular to the sample surface at an angle of 60°. The pass energies of the analyzer were 160 eV for survey spectra and 40 eV for high-resolution scans. The resolution of the spectrometer measured at a pass energy of 40 eV as an FWHM of the Ag 3d5/2 line was better than 0.75 eV. The binding energy scale of the spectrometer was preliminarily calibrated using the position of the peaks for Au 4f_7/2_ (83.96 eV), Ag 3d_5/2_ (368.21 eV), and Cu 2p_3/2_ (932.62 eV) core levels of pure metallic gold, silver, and copper with an uncertainty better than ±0.05 eV. The Kratos charge neutralizer system was used, and the spectra were charge-corrected to give the Al2p peak a binding energy of 74.4 eV, which is typical for aluminum in oxides, hydroxides, and oxyhydroxides [10]. Spectra were processed in CasaXPS software using the U2 Tougaard background subtraction. The O1s spectra were fitted with two GL(30) Gaussian-Lorentzian curves.

A Nicolet 6700 spectrometer (Thermo Scientific, Waltham, MA, USA) equipped with a mercury– cadmium–telluride (MCT) detector cooled with liquid N_2_ and a smart specular apertured grazing angle (SAGA) accessory were used to record IR reflectance spectra in the range of 4000 ÷ 650 cm^−1^. The angle of incidence was 80 degrees, and the diameter of the circular sampling area was 5 mm. The spectra were recorded at a resolution of 4 cm^−1^. All presented spectra were obtained by averaging 128 scans and were recorded at room temperature. All spectra were processed using OMNIC^TM^ software.

To characterize the wettability of superhydrophilic samples, we analyzed the shape of 10 mL distilled water droplets that had been deposited onto the surface. For the measurement of the contact angles and roll-off/sliding angles for hydrophobic and superhydrophobic samples, the homemade setup described in [11] was used. Each reported value was obtained by averaging over at least five droplets placed at different locations of the sample being tested for two different samples. 

The robustness of the surface layer of the samples subjected to thermocycling against abrasive wear was studied according to the ASTM F735 standard in an oscillating sand abrasion test [12]. Prior to the wear test, the samples were hydrophobized by the chemisorbed layer of fluorosilane as described in Section 2.3. That allowed us to characterize quantitatively the degradation due to abrasive wear by varying the contact and roll-off/sliding angles. To test the wear robustness, the sample was fixed at the bottom of the container and covered by a 12.5 mm layer of calibrated sand having a particle size of 0.5–0.8 mm. In the next step, the container was placed on a shaker (Heidolph Vibramax 100) with a vibration frequency of 1050 rpm and vibration amplitude of 3 cm. After 20 min of shaking, the sample was removed, thoroughly rinsed with distilled water, air-dried for 30 min at an ambient humidity of 40–50%, and the contact and roll-off/sliding angles for the water droplets were measured.

## 3. Results

**Figure 1:**
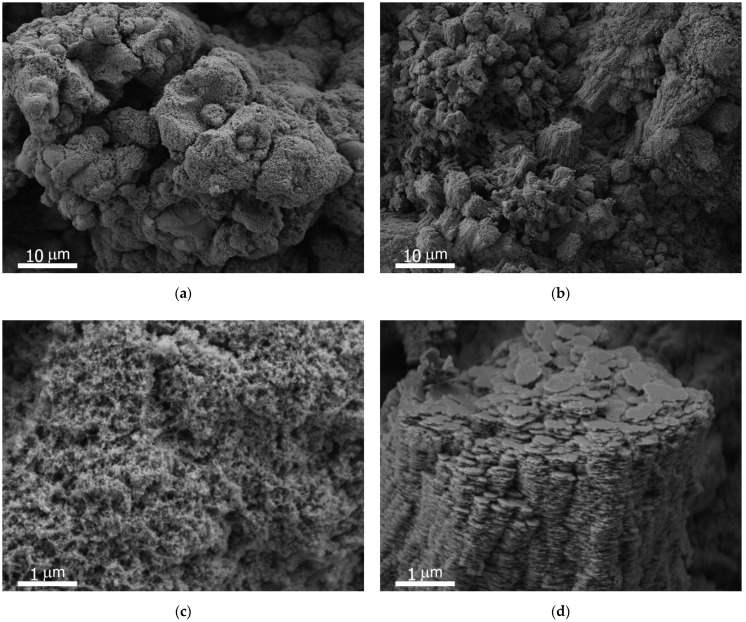
The SEM images of the sample’s surface at different magnifications before (**a**,**c**) and after (**b**,**d**) the thermocycling treatment. Despite the notable change in morphology, high surface roughness both prior to and after thermocycling leads to the preservation of superhydrophilic properties.

The SEM images of the sample’s surface before the beginning of the thermocycling treatment are shown in Figure 1a,c. The texture is characterized by hierarchical roughness, which formed due to material evaporation and the precipitation of metal nanoparticles formed in a laser plume during laser treatment. According to the Wenzel law for the wettability of intrinsically hydrophilic rough surfaces, such multimodal roughness should cause an essential decrease in the apparent contact angle for rough surfaces compared to flat ones. In particular, a sufficiently developed morphology on a hydrophilic metallic material will lead to a decrease in the apparent contact angle to zero, thus demonstrating superhydrophilicity.

**Figure 2:**
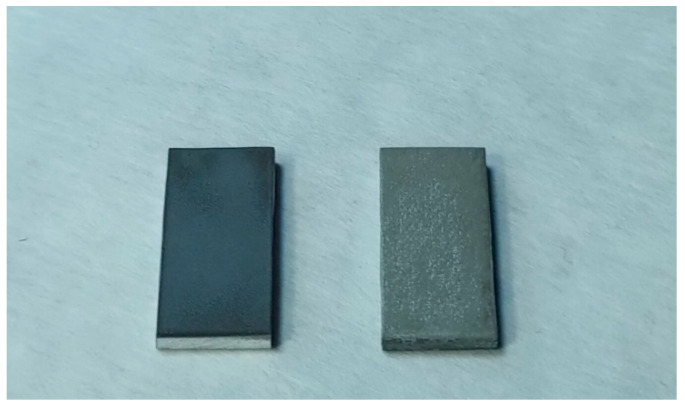
Photographic image of fresh superhydrophilic sample (**left**) and superhydrophilic sample after 53 thermal cycles (**right**).

An analysis of the wettability of the samples just after laser texturing showed the superhydrophilic state of the samples. Water droplets deposited onto the surface spread quickly and completely. It was expected that, due to the impregnation of the texture by a liquid, the subsequent freezing and melting of water during the cyclic temperature treatment of the sample should lead to heavy frosting and texture degradation, which should result in the fracture of the texture elements and loss of the superhydrophilic state. However, careful inspection of the water-spreading process during tens of thermal cycles did not reveal any compromised superhydrophilicity. At the same time, after a large number of cycles, a change in the sample appearance from dark gray to light gray with white spots was detected (Figure 2). For different samples, this threshold number varied from 30 to 50 freezing/melting cycles. This change in the sample’s surface color suggested the formation of a new phase as a result of the prolonged interaction of the surface aluminum oxide with water of varying temperature.

After the instant a new phase on the surface of the samples was detected, the thermocycling treatment was continued; however, with an increase in the number of cycles, the amount of the new phase did not visually change. It is also worth noting that the formation of a new phase did not affect the wettability of the samples by water; that is, the samples retained their superhydrophilic properties. In addition, the contact of such a sample with an abundant amount of water led to the transition of white microparticles from the surface to the aqueous phase.

To determine the phase transitions taking place in the surface layers of the laser-textured sample in contact with an aqueous phase, we performed studies of the surface layers using FE-SEM, EDX, Fourier-IR reflectance spectroscopy, and XPS. 

A comparison of SEM images of the laser-textured surface before and after thermocycling indicated a drastic change in morphology. Thus, for the initial state of the samples, the texture was formed by ablation grooves (not shown here), that were decorated by micro- and nanoaggregates and individual nanoparticles deposited onto the surface from the laser plume during laser ablation (Figure 1a,c).

However, after thermocycling, a transformation of the morphology of aggregates was observed with the replacement of nanoparticles by the nanoplatelets as the main texture element. The newly formed morphology of the surface was represented by the columnar texture with densely packed flat platelets with lateral sizes of 200–300 nm and a thickness less than 10 nm (Figure 1b,d). 

The detailed EDX analysis of the elemental composition of the surface layer of the AMG2 alloy after laser texturing using the laser processing parameters used in this study [8] showed that the uppermost layer consisted mainly of aluminum, oxygen and a small amount of nitrogen detected in the deeper surface layers. As for the phase composition, it was mainly composed of γ-Al_2_O_3_. 

**Figure 3:**
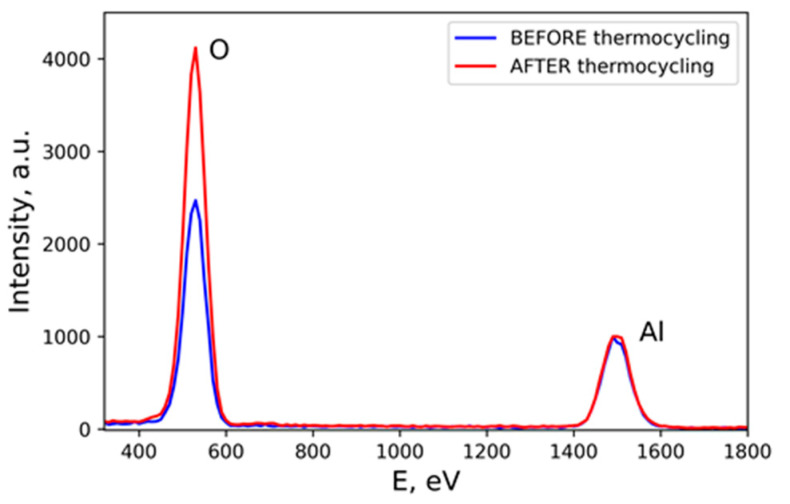
Typical EDX spectra of the samples before and after thermocycling.

A comparison of the EDX spectra of the sample before and after thermocycling as shown in Figure 3 indicated a notable increase in oxygen content. A rough estimate determined that this increase was more than 1.5–1.7 times.

**Figure 4:**
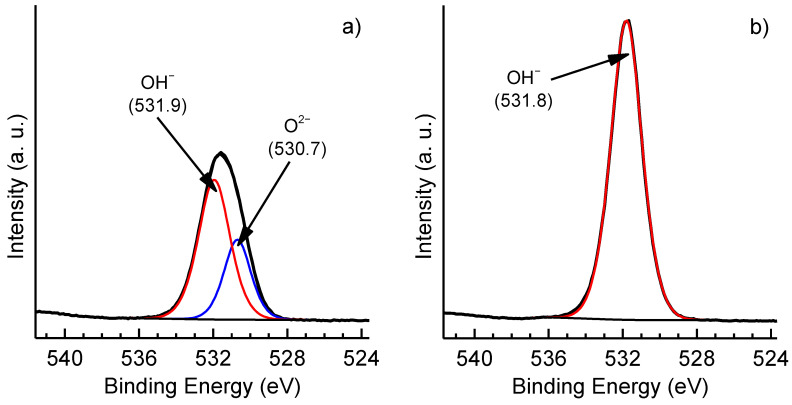
O1s XPS spectra of uncycled (**a**) and thermally cycled (**b**) samples. Each spectrum was normalized to the area of the corresponding Al2p spectrum.

XPS analysis confirmed the conclusions based on EDX data: after thermal cycling, the surface oxygen-to-aluminum ratio increased by about 1.5 times from 2.1 to 3.0. The Al2p spectra could not be used to unambiguously distinguish aluminum oxides, hydroxides, and oxyhydroxides because of similar Al2p binding energies [10] and uncertainty in charge referencing. At the same time, the O1s spectra of these compounds demonstrated larger chemical shifts. The O1s spectrum of the uncycled sample (Figure 4a) showed a broad peak that fitted with two components centered at 530.7 and 531.9 eV. These components were respectively attributed to lattice oxygen in alumina (O^2−^) and oxygen in hydroxyl groups (OH^−^) bonded to aluminum atoms [10]. A broad O1s spectrum is typical of aluminum oxyhydroxides [10,13]. It is worth noting that oxygen from surface contaminations may also have contributed to the O1s spectrum, especially the higher binding energy component. After thermal cycling, the lattice oxygen component completely disappeared and the O1s spectrum narrowed significantly (Figure 4b), which, along with the increase in oxygen content, attested to the formation of aluminum hydroxide Al(OH)_3_ on the surface.

**Figure 5:**
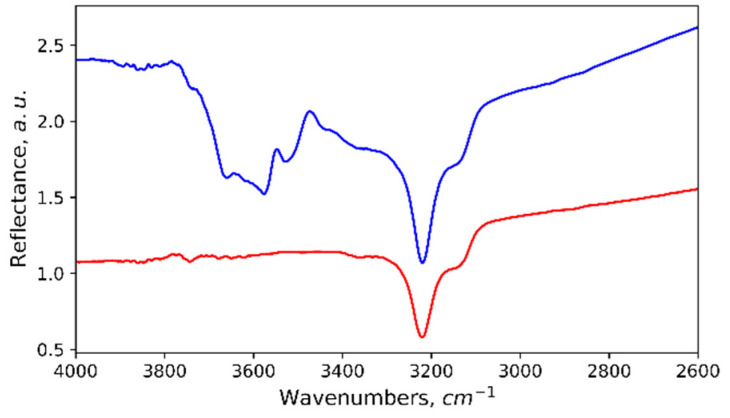
Fourier-IR spectra of uncycled (red) and thermally cycled (blue) samples.

The presence of hydroxides on the surface of our sample was confirmed by the reflectance Fourier-IR spectra (Figure 5). Active bands visible on the spectra in the range of 3850–2800 cm^−1^ were associated with O–H vibrations in hydroxides, the numerous bands in the range of 3700–3400 cm^−1^ were assigned to hydroxyl stretching vibrations in bayerite or gibbsite [14,15], while the band in the 3300–3000 cm^-1^ range was associated with O–H vibrations in boehmite [16]. A comparison of the reflectance spectra for the initial state of the sample just after laser texturing, washing and drying with the spectra after thermal cycling indicated that the boehmite phase seemingly resulted from laser-treated surface interaction with atmospheric water or was formed during sample washing in water using an ultrasonic bath. This phase survived during the thermocycling of the sample, and its quantity even grew slightly as may be concluded from the increase in the 3225 cm^−1^ band area.

Unfortunately, the proximity of the maxima of the main bands of the O–H stretching vibrations for gibbsite and bayerite in the 3700–3400 cm^−1^ range made it impossible to determine unambiguously which of the hydroxide phases prevailed. At the same time, being characterized by a layered structure, both phases were close to each other in mechanical properties.

Now, let us consider the resistance of hydroxides formed during thermal cycling to wear abrasion in the sand oscillating test. It is worth noting that to characterize the evolution of the sample’s morphology in the course of abrasion quantitatively, it was decided to hydrophobize the surface before the abrasion experiment. This method allowed accurate measurement of variations in the contact and roll-off water angles on surfaces induced by the degradation of the hierarchical texture upon abrasion. The wettability characteristics presented in Table 1 for the sample after thermocycling with 101 cycles indicated that the initial superhydrophilic state of the sample had been replaced by a superhydrophobic state after deposition of fluorooxysilane onto the texture formed after thermocycling. 

It was interesting to compare the impact of the surface morphology on the wettability of two types of samples. We present in Table 1 the contact and roll-off angles for the sample after laser texturing followed by deposition of flurosilane and after laser texturing followed by thermal cycling and hydrophobization by fluorosilane. Although the contact angles were nearly the same for these two samples, the roll-off angle for the sample subjected to thermocycling was shown to be 3 times higher with notably larger scattering across the sample. Analysis of the wettability evolution during 20 min of the wear abrasion test for both samples (Table 1) indicated notably lower mechanical resistance after thermal cycling in comparison to the initial sample after the laser treatment.

## 4. Discussion

It has been discussed in the literature that aluminum oxide surfaces are stable in an aqueous environment with a wide range of pH from 3 to 9 [17]. However, experimental studies of η-Al_2_O_3_ and γ- Al_2_O_3_ dispersed in aqueous media having different pH indicate oxide chemical activity and transformation to hydroxide phases [18,19,20,21,22]. This transition takes place in both the liquid aqueous phase and in a humid atmosphere [19,23]. The literature shows that, in the presence of water, hydroxides demonstrate a higher thermodynamic stability than alumina, supported by both experiment and thermodynamic calculations [18,19,22]. The reaction of alumina particles with water might result in different products such as bayerite β-Al(OH)_3_, gibbsite α-Al(OH)_3_, or boehmite AlOOH, depending on temperature, pH of the aqueous phase, and type of oxide [18,19,20,21,22,23,24]. For example, at room temperature and low pH the gibbsite phase prevailed, while a high pH facilitated bayerite formation [18,19,20,21,22]. Increased temperatures promoted the formation of boehmite or pseudo-boehmite [25,26] when in contact with water on a textured aluminum surface. In our experiments, hierarchical surface morphology was constituted by nanoparticle aggregates having a highly developed surface, which came in contact with cold/room temperature water in a homogeneous (Wenzel) wetting regime. EDX, SEM, Fourier-IR reflectance spectroscopy and XRD data as presented above showed the formation of either gibbsite or bayerite. The complex surface morphology made it difficult to differentiate between these two hydroxides with the same composition but different structures: different relative stackings of the same unit layers. For a better understanding of the phase type for the hydroxide that formed upon temperature cycling, we measured the pH variation of the aqueous phase in contact with our samples during 5 thermocycles. Since we changed the water inside the polypropylene containers with the sample every 5 cycles, as described in the experimental section, the duration of surface contact with the same water bath exceeded 900 min. Such a protocol was selected to mimic the contact of a superhydrophilic surface with atmospheric precipitation under outdoor conditions. It was found that the pH of the aqueous medium for the freshly textured sample quickly deviated from the value of pH 5.6 ± 0.1 characteristic of water at the beginning of this experiment. After one cycle, the alkalinity of the medium increased to pH 6.6±0.2, while after 5 cycles it reached pH 7.2 ± 0.2. On the one hand, this increase in the alkalinity of the aqueous phase was related to the oxidation of the sample surface, where nanoparticles of alumina decorated the substrate, containing patches of nonoxidized aluminum. For the nonoxidized aluminum patches, the following reaction took place:

2Al + 6H_2_O = 2Al(OH)_3_ + 3H_2_↑.

For the superhydrophilic sample in contact with the aqueous phase for tens of cycles, the rate of pH variation was somewhat lower, from pH 5.6 ± 0.1 to 6.8 ± 0.2. The chemical interaction of alumina with water, characterized by the negative value of the free energies of hydration [19], is described by the reaction:

Al_2_O_3_(s) + 3H_2_O(l) = 2Al(OH)_3_(s)

Notations (s) and (l) denote the solid or liquid state of the reaction components. In the literature, three different transformation mechanisms from the oxide to hydroxide phase were considered [18,27]. The first one was associated with alumina surface hydration through hydrolysis of Al–O-Al surface bonds and the formation of Al-OH. It was expected that after this mechanism the transformation of the texturing elements composed of alumina should result in the preservation of the macro- and microsurface texture. The second mechanism was based on the dissolution of oxide, followed by the precipitation of the hydroxide from the saturated or supersaturated dispersions of hydroxide nuclei [18]. This mechanism resulted in the formation of a new poorly ordered type of texture atop our hierarchically rough samples. Finally, according to [27], the third mechanism related the conversion of alumina to aluminum hydroxide through the occupation of the vacancies left due to inward oxygen diffusion by OH^−^ ions. Following this mechanism, the preservation of initial micro and macrotexture was highly likely. 

From the SEM pictures of our samples after prolonged thermocycling (Figure 1b,d) it can be concluded that the morphology of the initial sample’s surface was partially inherited, while microaggregates of nanoparticles were replaced by a layered texture formed by ordered plates. It looks like the hydroxide was formed by the joint action of all mentioned mechanisms of formation. The substitution of the oxide texture by the hydroxide caused a weakening of the mechanical resistance. In our recent studies [8,20], grain refinement, hardening, and enrichment of the surface layer with nano-inclusions of aluminum oxynitride during laser processing provided a significant increase in the resistance of the surface layer to abrasion. Thus, the laser-processed samples demonstrated good mechanical stability. For the hydroxide, the crystalline structure of gibbsite and bayerite differed only in the packing arrangement of the Al–O–Al layers [3], which were bound to each other by a network of hydrogen bonds. The weakness of the H bonds between the layers caused easy destruction of the layer network for both polymorphs.

It should be noted that in this study we chose the AMG2 aluminum–magnesium alloy because it is widely used in the industry. This alloy has quite a low bulk Mg concentration of 2.9%, however, the surface concentration of magnesium after laser processing was even lower. The absence of a peak corresponding to Mg on the EDX spectra (Figure 3) led to the conclusion that the Mg concentration in surface layers was lower than 0.2–0.3%. This result, known in the literature, was not surprising and can be explained by the high volatility (in comparison with Al) of magnesium during laser-induced vaporization. Thus, the effect of additional alloy compounds on the processes considered in this manuscript is negligible.

## 5. Conclusions

Our studies showed that the superhydrophilic samples of aluminum alloy with the hierarchical alumina texture are not stable when in contact with water and cyclic variations from room temperature to low negative values. Chemical interaction of the hierarchically rough alumina with water molecules caused the oxide to be replaced by polymorph crystalline phases (gibbsite or bayerite). Although the preservation of the hierarchical roughness during such substitution allowed the retention of superhydrophilic properties, it was accompanied by a notable compromise in the mechanical resistance. In particular, the poor mechanical properties of aluminum-layered hydroxides led to weaker abrasive resistance. Thus, in contrast to the superhydrophobic laser-processed samples, which demonstrated very high durability in various atmospheric conditions [28], the superhydrophilic samples showed chemical transformation and mechanical degradation when in contact with water under cyclic temperature variation. Therefore, despite their potential anti-icing properties, superhydrophilic aluminum surfaces cannot be considered as an attractive way for industry to combat icing in open atmospheric conditions.

## Figures and Tables

**Table 1:** The impact of sand abrasion test on the wettability of uncycled and thermally cycled hydrophobized samples.

Sample		Contact Angle °	Roll-Off Angle °
Laser-textured sample subjected to fluorosilane deposition	As prepared	171.5 ± 0.8	2.0 ± 0.6
	After 20 min of abrasion load	159.8 ± 2.5	17.1 ± 1.8
Laser-textured sample, subjected to thermal cycling and fluorosilane deposition	As prepared	171.2 ± 0.7	6.2 ± 2.3
	After 20 min of abrasion load	146.3 ± 1.7	43.2 ± 9

## Data Availability

Not applicable.
